# Sarcoidosis-induced pericarditis in a patient with portopulmonary hypertension: a case report

**DOI:** 10.4076/1757-1626-2-8640

**Published:** 2009-08-18

**Authors:** Olga Giouleme, Panagiotis Anagnostis, Kalliopi Patsiaoura, Themistoklis Vasiliadis, Nikolaos Grammatikos, Nikitas Kakavas, Alexander Mpoumponaris, Nikolaos Eugenidis, Elias Basayannis

**Affiliations:** 1 Division of Gastroenterology, Department of Internal Medicine, Aristotle University, Hippokration Hospital49 Konstantinoupoleos Str 54642, ThessalonikiGreece; 2 Cardiology, 2^nd^ Propedeutic Department of Internal Medicine, Aristotle University, Hippokration Hospital49 Konstantinoupoleos Str 54642, ThessalonikiGreece

## Abstract

Portopulmonary hypertension is a rare and severe complication of patients with cirrhosis. Sarcoidosis, a disease of unknown etiology, is also a cause of pulonary hypertension and right heart dysfunction. We report the case of a 51-year-old male patient, suffering from cirrhosis due to Wilson’s disease, portal hypertension and pulmonary hypertension (PH), who developed severe pericarditis. Wilson’s disease was diagnosed 8 years before his last admission to our hospital and was being successfully treated with D-penicillamine. PH was recognized 2 years before admission and being treated with bosentan. The patient complained for dyspnea at rest and the 2D echocardiogram revealed a significant amount of pericardial fluid. All other causes of acute pericarditis were excluded and his laboratory, imaging and histopathological investigation showed evidence of sarcoidosis. He underwent a therapy with corticosteroids (methylprednisolone) and his follow-up examination showed remarkable decrease of the levels of mean pulmonary artery pressure and pericardial fluid.

## Introduction

Portopulmonary hypertension (PPHT) is a pulmonary vascular consequence of advanced liver disease. Some studies suggest that the frequency of PPHT in cirrhotic patients is about 6%. The pathogenesis of PPHT is suggested to be based on an increased flow or volume phenomenon, secondary to the hyperdynamic circulation of cirrhosis and vasoconstriction due to an increase in vasoconstrictive substances entering the pulmonary circulation from the hepato-splanchnic vascular bed, such as endothelin-1 and others [1].

Pulmonary hypertension (PH) is also a well-known complication of sarcoidosis. In patients with sarcoidosis the prevalence of PH and right heart dysfunction has been reported to range from 4% to 28%. In most cases, sarcoidosis-related PH is mild to moderate or is manifested only with exercise. Severe PH has been described in sarcoidosis usually in association with severe fibrotic parenchymal disease. It can also occur in the absence of pulmonary fibrosis by means of granulomatous vasculitis of the pulmonary vessels or plexiform pulmonary arteriopathy or extrinsic compression of large pulmonary arteries by mediastinal or hilar adenopathies. Uncommonly, portal hypertension secondary to liver sarcoidosis can also induce PH [2,3].

## Case presentation

A 51-year-old Caucasian male was admitted to our department complaining for dyspnea, for about 20 days before admission, combined with fatigability, without chest pain, cough, fever or other relative symptoms. He had been hospitalized in our department because of jaundice and signs of hepatic failure 8 years ago. At that time the investigation had led to the diagnosis of Wilson’s disease. D-penicillamine was administered and the patient responded impressively. Since then he was being followed-up at regular intervals. Six and a half years later, he was admitted again due to fatigue. He was diagnosed as having PH on 2D echocardiography and started therapy with bosentan (firstly 67.5 mg bid and then 125 mg bid) and sildenafil (25 mg tid and then 50 mg tid). Due to his clinical and echocardiographic improvement, 3 months later, he was treated only with bosentan at the same dose until his last admission.

On physical examination he had peripheral edema, inflated jugular veins and vascular spiders, a systolic murmur of the tricuspid valve, elevation of S_2_ and split of S_2_ at the focus of pulmonary valve and splenomegaly. The electrocardiography revealed right axis deviation and right bundle branch block. The chest radiograph showed a “water bottle” configuration of the cardiac silhouette and ambilateral enlargement of the pulmonary hili, and the 2D echocardiography revealed presence of significant amount of pericardial fluid, as well as increased dimensions of right cavities and mild regurgitation of the tricuspid valve. The right ventricular systolic pressure (RVSP), as estimated by echocardiography, was 75-80 mmHg (normal <25 mmHg).

Laboratory testing on admission revealed leucopenia (WBC: 3,600/μL) and thrombocytopenia (PLT: 83,000/μL). Cirrhosis was well compensated: INR: 1.33, albumin: 4.6 g/dL. The other liver function tests were normal, as was his renal function.

The diagnostic approach to our patient was headed towards the etiology of pericarditis. Viral markers [Hepatitis B and C, Human Immunodeficiency Virus (HIV), Ebstein-Barr, Herpes Simple, Cytomegalovirus, Echo, Parvo, Coxsackie and Arbo] as well as investigation for Mycoplasma Pneumoniae, Chlamydia and Coxsiella Burnetti, were negative. His autoimmune profile, tumor markers and thyroid function were normal. Finally, a tuberculin skin test (Mantoux) was performed, which was positive (infiltration of 15 mm).

**Figure 1.**
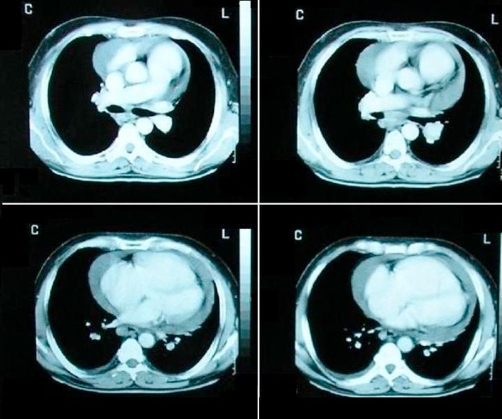
Pericardial fluid (CT of the thorax).

Regarding the patient’s situation and the positive Mantoux-test, a Computerized Tomography (CT) of the thorax (Figure 1) and abdomen was performed, which showed, apart from a significant amount of pericardial fluid, enlarged lymph nodes of the anterior and right posterior mediastinum, left hilum, carina and the left part of the pulmoaortic window. The CT of the abdomen revealed liver cirrhosis, splenomegaly and dilatation of the portal and splenic vein. As a result of these, we decided to check the levels of the serum angiotensin-converting enzyme (ACE), which were high: 68IU/L (normal range: 0-52 IU/L).

The patient’s initial treatment was to decrease the dose of bosentan until discontinuation (considering that bosentan causes peripheral edemas) but no improvement was noticed. On the 10th day of hospitalization, pericardiocentesis was performed. The pericardial fluid was sanguineous, about 1,200cc, exudate, with about 100 WBC (66.1% neutrophils, 21% lymphocytes, 4.8% monocytes and 8.1% eosinophils) and haematocrit 0.7%. Specimens of pericardial fluid were obtained for culture [aerobe and non aerobe microorganisms, as well as Mycobacterium tuberculosis (Tb)]. All were negative, as was the PCR test for Tb. The cytological analysis of the pericardial fluid did not show evidence of malignancy. Finally, no specific granulomatous inflammation or malignancy was noticed on pericardial biopsy.

**Figure 2.**
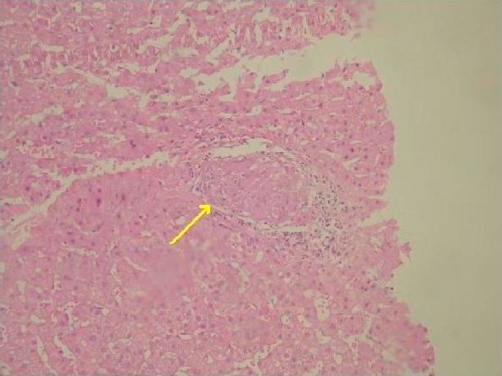
Hepatic parenchyma with the presence of a non caseating granuloma (arrow) (AEx100).

On the 11^th^ day, the patient restarted therapy with bosentan (67.5 mg bid) and on the 15^th^ day sildenafil was added (25 mg bid) in combination with colchicine (0.5 mg bid). In order to check the liver’s condition, respectively to Wilson’s disease, the patient underwent a liver biopsy. On histological examination most of the portal tracts were enlarged due to fibrosis and inflammatory infiltration, mainly with lymphocytes. Periportal hepatitis and portal-portal ligation was also seen. In the parenchyma, a granuloma was revealed, without central necrosis, with the presence of multinucleated giant cells. The histological diagnosis was consistent with granulomatous hepatitis (Figure 2). Consequently, the diagnosis was headed towards sarcoidosis. Therefore, a scintiscan with gallium (Ga^67^) was performed, which was indicative of sarcoidosis.

On the 18^th^ day, methylprednisolone was administered (16 mg tid), in combination with bosentan, sildenafil and colchicine, on the doses mentioned above. D-penicillamine was replaced by zinc acetate, considering that the role of D-penicillamine in terms of inducing granulomatous hepatitis and generation pericardial fluid was indefinite. As a result of this, the levels of copper of 24-hour urine were significantly reduced [from 700 μg/24h to 90 μg/24h (target during maintenance of zinc therapy is 50-125 μg/24h)].

One month later, the echocardiograph showed significant decrease in the amount of pericardial fluid, but unremarkable reduction of the levels of RVSP, although the patient was not complaining for dyspnea any more. Nonetheless, 2 months later, he had only a slight quantity of pericardial fluid and the estimated RVSP was 40-45 mmHg. Furthermore, there was also a decrease in the levels of the serum ACE on the 6-month-follow-up down to 31 IU/L. In the following months the dose of methylprednisolone was tapered to 24 mg per day and 1 year later (after 2 relapses of pericardial infusion successfully treated with an increase of methylprednisolone to 32 mg per day with gradual tapering), he is being treated in a maintenance dose of 16 mg per day in combination with bosentan 125 mg bid and sindenafil 25 mg bid. The mean pulmonary arterial pressure (MPAP) performed on right heart catheterization on 1-year-follow-up in another hospital was 60-70 mmHg.

## Discussion

Cardiac sarcoidosis is often overlooked because of its subclinical disease progression. Approximately 30% of patients with systemic sarcoidosis have granulomas in the myocardium and 5% have sings of cardiac affection. The pericardium can be involved in sarcoidosis with and without clinically evident myocardial involvement. Manifestations of pericardial sarcoidosis include symptomatic or asymptomatic pericardial effusion, with or without cardiac tamponade, and chronic constrictive pericarditis. Sarcoidosis of the pericardium has a good prognosis in the absence of clinically detectable myocardial involvement [4].

Although corticosteroids remain the mainstay of treatment, there is little evidence for the optimal initiation, dosage or duration of therapy. Doses of 60-80 mg of prednisone daily are generally prescribed initially. In some studies, the starting dose of 30 mg/day was proven sufficient to improve prognosis. Patients should be re-evaluated after 2-3 months, and if the disease is responding, the dose must be tapered gradually to a maintenance level of 10-15 mg per day over a period of six months. If serial evaluations reveal that the disease is controlled, corticosteroids may be tapered further and eventually discontinued. Prerequisites for steroid taper or withdrawal include absence of disease activity, confirmed by radionuclide imaging and by serum determinations of ACE if values were initially elevated at time of diagnosis. Alternative agents such as antimalarials, methotrexate and azathioprine may be given to patients who do not respond to corticosteroids or who cannot tolerate their side effects [5-7].

In addition, the patient’s positive Mantoux test and mediastinal lymphadenopathy set Tb in terms of differential diagnosis. However, there was much more evidence towards sarcoidosis, (regarding especially the patient’s liver biopsy and the negative PCR test for Tb), which forced us to initiate corticosteroid therapy. The long term administration of methylprednisolone did not induce manifestation of Tb.

The other therapeutic agent for our patient is bosentan, an endothelin receptor antagonist, which has been shown to be beneficial in the treatment of primary pulmonary hypertension, but its efficacy in secondary pulmonary arterial hypertension (SPAH) has not been established. SPAH is an adverse outcome of a variety of systemic disorders such as collagen vascular diseases, chronic thromboembolism, human immunodeficiency virus, portopulmonary hypertension, sarcoidosis and other diseases [8,9].

In the literature there are few published case reports showing the safety and efficacy of bosentan in patients with PPHT. Our cirrhotic patient was initially treated successfully with bosentan for about one year before his last admission. One potential explanation for the effectiveness of bosentan in such cases derives from the fact that increased hepatic expression of endothelin with hepatosplachnic spillover is one putative mechanism for the development of portopulmonary hypertension [10,11].

It must also be mentioned that D-penicillamine is a very rare cause of drug-induced granulomatous hepatitis (GH). We culled only one case report from the literature [12]. GH has been attributed to a variety of drugs such as: allopurinol, aspirin, diazepam, isoniazide, phenitoin and sulphonamides. Other causes of GH incude tuberculosis (the commonest), sarcoidosis, leprosy, histoplasmosis, brucellosis, lymphoma and malignant granuloma [13,14]. The associated lesions of liver injury suggesting a drug etiology include significant tissue eosinophilia. Eosinophils are rare to absent in tuberculous hepatic granulomas and, when present in significant numbers, militate strongly against sarcoidosis [15,16]. No eosinophils were described in our patient’s biopsy. To the best of our knowledge, the co-existence of Wilson’s disease has never been reported in the literature. Furthermore, pericardial effusion along with PH has a very poor prognosis [17-19] and has been described in few conditions, except for sarcoidosis, such as sickle cell disease [19], HIV infection [20], primary Sjögren's syndrome (usually with primary biliary cirrhosis and cryoglobulinemia) [21], systemic sclerosis [22], systemic lupus erythematosus [23], Behcet's disease [24]. Pericardiocentesis and percutaneous balloon pericardiotomy appear to be a reasonable therapeutic approach, although it is characterized by high mortality [18].

## Conclusion

In conclusion, we described a patient who, until his last admission to out department, was thought to suffer from SPAH, induced only by liver cirrhosis due to Wilson’s disease. His further investigation on occasion of acute pericarditis provided evidence of sarcoidosis, as another factor for SPAH. This was indicated by his rapid improvement with corticosteroids, respectively to pericarditis, on the one-month follow-up visit, and by the gradual decrease in the levels of mean pulmonary arterial pressure, on the three-month follow up visit. We also hypothesize that the combination of bosentan with sildenafil led to further improvement of pulmonary hypertension.

## Abbreviations

ACE: angiotensin-converting enzyme
CT: computerized tomography
GH: granulomatous hepatitis
HIV: human immunodeficiency virus
MPAP: mean pulmonary arterial pressure
PCR: polymerase chain reaction
PH: pulmonary hypertension
PLT: platelets
PPHT: portopulmonary hypertension
SPAH: secondary pulmonary arterial hypertension
Tb: tuberculosis
WBC: white blood cells
